# Frequency and features of medical emergencies at a teaching dental hospital in Saudi Arabia: a 14-year retrospective observational study

**DOI:** 10.1186/s12873-024-00957-4

**Published:** 2024-03-13

**Authors:** Maisa O. Al-Sebaei

**Affiliations:** Department of Oral and Maxillofacial Surgery, King Abdulaziz University - Faculty of Dentistry, 21589 Jeddah, PO Box 80209, Saudi Arabia

**Keywords:** Retrospective studies, Emergencies, Dental Clinics, Anesthesia, Local, Emergency Service, hospital, Dentistry, Hypoglycemia, Syncope, Anxiety

## Abstract

**Background:**

This study aimed to determine the nature, frequency, and characteristics of medical emergencies occurring at the King Abdulaziz University Dental Hospital.

**Materials and methods:**

The incident reports of medical emergencies recorded at the King Abdulaziz University Dental Hospital from January 2008 to December 2022 were retrospectively reviewed. The annual/overall incidence of medical emergency events was calculated per 100,000 patients. The following characteristics of the patients/events were evaluated: age, gender, operator, procedure, location, timing of treatment, administration of local anesthesia, past medical history, symptoms, diagnosis, outcome, and disposition. Multivariable logistic regression models were used to investigate the associations of these characteristics with two outcomes: syncope and transfer to the emergency room (ER).

**Results:**

The incidence of emergency events was 17.4 per 100,000 patients. Syncope and hypoglycemia were the most common emergencies. Most incidents recovered, with only 13% requiring transfer to the ER. Undergoing no procedure and American Society of Anesthesiologists (ASA) class 2,3 were associated with syncope. Undergoing a general dental procedure, ASA class 2,3, and a diagnosis other than hypoglycemia and syncope were associated with transfer to the ER.

**Conclusions:**

The incidence of medical emergencies was low. Dental practitioners need to remain aware of the contributing factors, such as past medical history and anxiety, but medical emergencies can occur in healthy individuals as well. Preparation of the dental office, training of the personnel, and proper recording of the events are essential components of a well-established medical emergency protocol in dental institutions.

**Supplementary Information:**

The online version contains supplementary material available at 10.1186/s12873-024-00957-4.

## Background

Medical emergencies can occur in any healthcare setting, including the dental office. These events may occur before, during, or after treatment. Between 50 and 70% of all dental practitioners witness at least one medical emergency event each year, while more than a quarter could encounter multiple events [1]; retrospective surveys among dental practitioners report a prevalence between 0.5 and 2 events per dentist per year [2, 3]. The incidence of medical emergencies in the dental setting has been described in a few studies only. In a retrospective study from a Japanese university dental hospital, the incidence of medical emergencies was 3.7 per 100,000 patients visiting the dental outpatient department [4]. In another retrospective study from an Australian dental school, the reported incidence of medical emergencies in dental clinics was 37 per 100,000 outpatients [5]. However, the incidence of medical emergency events in dental offices of academic dental institutions of the Middle Eastern countries remains unreported.

Medical emergencies can be mild and self-limiting or serious and life-threatening. Preparedness for any medical emergency and understanding its cause, diagnosis, and management is vital, especially when patients undergo treatment in large teaching dental hospitals, such as our institution with 361 dental operatories and clinics.

A retrospective analysis of records and databases is integral to improving the quality of services and outcomes related to medical emergency events occurring in the dental office.

However, in this context, most studies reported in the literature are survey-based [6–9]; only seven retrospective studies were found, that used patient databases and/or patient records and involved dental teaching hospitals or dental school settings [4, 5, 10–14]. None of these seven retrospective studies were from the Middle East; one study each was reported from Japan [4], the United Kingdom [12], and Australia [5]; four studies were from the United States [10, 11, 13]. Except for the studies by Kim et al. (*n* = 586, study duration = 10 years) [13] and D’Innocenzo et al. (*n* = 250, study duration = 12 years) [14], most other studies had a study duration of less than 10 years and a relatively small sample size, with some of them reporting emergency incidents in double digits only. Hence, the characteristics of medical emergencies arising in the dental offices seem to be inadequately described, particularly in the Middle East.

Therefore, this study aimed to determine the nature, frequency, and characteristics of medical emergencies occurring at the King Abdulaziz University Dental Hospital (UDH) over a 14-year period. To our knowledge, this is the longest retrospective study of medical emergencies in the dental office in the literature and the first in Saudi Arabia. The data in the incident reports at our institution were documented by the emergency response team, operator, and supervising faculty, which assures their accuracy and consistency.

## Materials and methods

### Study design and ethical approval

This retrospective study was approved by the Research Ethics Committee of King Abdulaziz UDH (#044 − 15). A waiver for patient consent was obtained for this study owing to the retrospective nature of chart review without further patient interaction. The incident reports of medical emergencies recorded at King Abdulaziz UDH from January 2008 to December 2022 were reviewed.

### Study setting and population

The medical emergency incident reports included in this study described medical emergencies occurring in patients seeking treatment at the UDH. The inclusion criteria were: Medical emergency events occurring within the brick-and-mortar buildings of the facility; adult and pediatric patients; those occurring in both clinical and non-clinical locations, such as the radiology area, and waiting rooms; and events occurring before, during and after dental treatment. The exclusion criteria were as follows: incomplete reports; emergencies managed without calling the medical emergency response team; non-urgent and chronic complaints that did not require immediate medical attention; minor cuts that required only first-aid; those occurring outside the building; and non-patient medical emergencies, such as those arising among students, employees, faculty, or by-standers.

### Study protocol and measures

When a medical emergency occurs at the King Abdulaziz UDH, a protocol is followed to summon the medical emergency response team (MERT) by dialing an in-house number. This team is comprised of a Registered nurse (RN), an emergency medical technician (EMT), and an on-call dentist trained in in management of medical emergencies. The members of the team are all licensed by the Saudi Commission for Health Specialties (SCFHS) and hold certificates in Basic Life Support (BLS). Additionally, the on-call dentist is certified in Advanced Cardiac Life Support (ACLS). They all receive an intramural comprehensive training focused on the management of medical emergencies in the dental office. This training module is a well-established and structured mandatory annual program at the UDH that includes hands-on exercises, a didactic component, and role-play simulations [15]. The team members actively participate in programs, where they receive training in the development of teamwork, clinical, and communication skills.

The involved student or faculty member, the RN, and the emergency response faculty member write a detailed account of the incident. This incident report captures demographics, date, time, location, type of dental treatment performed at the time of the incident, timing of the incident with regards to dental treatment and local anesthesia, level of the operator, past medical history, American Society of Anesthesiologists classification (ASA) vital signs, incident details, diagnosis, management performed by the MERT, outcome, and disposition. These reports were reviewed and tabulated in an Excel sheet [Excel version 16.66.1, Microsoft Inc., USA].

### Statistical analysis

Descriptive statistics were used to summarize the characteristics of the patients, including age, gender, operator, procedure, location, timing of treatment, administration of local anesthesia, past medical history, symptoms, diagnosis, outcome, and disposition. The data were presented as means and standard deviations (SD) for continuous variables and as frequencies and percentages for categorical variables.

The annual incidence of medical emergency events was calculated per 100,000 patients by dividing the number of events in the given year by the number of patients visits in that year and multiplied by 100,000. The overall 14-year incidence of events was calculated by dividing the total number of events by the total number of patients visits and multiplied by 100,000.

Multivariable logistic regression models were used to investigate the associations between several predictor variables and two outcomes: syncope and transfer to the emergency room (ER). The predictor variables included age, gender, operator level, procedure performed, past medical history, use of local anesthesia and diagnosis. In the first multivariate logistic regression model with syncope (Yes/No) as the outcome, an odds ratio (OR) > 1 was associated with a higher likelihood of syncope. In the second multivariate logistic regression model that investigated the outcome of transfer to the ER (Yes/No), the diagnosis variable was grouped into four categories to simplify the model: Syncope, Hypoglycemia, Respiratory (asthma and hyperventilation) and Others (including seizure, chest pain, panic attack, hypertensive crisis, suspected cardiovascular accident, sodium hypochlorite accident, allergy, partial airway obstruction and dehydration). An OR > 1 was associated with a higher likelihood of transfer to the ER.

ORs and 95% confidence intervals (CIs) were calculated and presented for each predictor variable. All statistical analyses were performed using R statistical software, version 4.2.2 (R Core Team, 2022). All statistical tests were two-sided, and *p*-values < 0.05 were considered statistically significant.

## Results

### Characteristics and frequencies

A total of 300 patients were included over the 14-year study period; the total number of visits was 1,722,252. The patients’ age ranged from 6 to 83, with a mean of 34.2 (SD = 14.2); approximately two-thirds were female (*N* = 198, 66%). Most patients were healthy (ASA 1) (*N* = 210, 70%); only 30% had a positive past medical history (*N* = 90). The most common procedure was restorative or prosthodontic (35%) followed by dental extraction or surgery (30.7%). The patients’ demographics are summarized in Table 1.

**Table 1:** Demographics and characteristics of the study participants and medical emergency events

Study Participant Characteristics	***N*** = ***300***^***1***^	Emergency Event Characteristic	***N*** = ***300***^***1***^
**Age**		**Local Anesthesia**	
Mean (sd)	34.21 (14.17)	Yes	229 (76.3)
Median (Min, Max)	32 (6, 83)	No	71 (23.6)
**Age Group**		**Timing of local Anesthesia**	
<18	23 (7.7)	During Local anesthesia	3 (1.0)
18–30	119 (39.7)	After Local anesthesia	226 (75.3)
31–40	66 (22.0)	No Local anesthesia	71 (23.7)
41–60	80 (26.7)	**Timing of the Event**	
61+	12 (4.0)	Before procedure	118 (39.3)
**Gender**		During procedure	98 (32.7)
Male	102 (34.0)	After procedure	84 (28)
Female	198 (66.0)	**Operator**	
**PMH**		Undergraduate	184 (61.3)
No	210 (70.0)	Postgraduate	33 (11.0)
Yes	90 (30.0)	Intern	31 (10.3)
**Procedure**		General Practitioner	33 (11.0)
Restorative/Prosthodontic	105 (35.0)	Faculty	11 (3.7)
Extraction/surgery	92 (30.7)	None	8 (2.7)
Endodontic	52 (17.3)	**Location**	
No Procedure	23 (7.7)	Clinic area	275 (91.7)
Examination	10 (3.3)	Waiting room/lobby/washroom	21 (7.0)
Scaling	6 (2.0)	Radiology/labs	4 (1.3)
Pediatric	6 (2.0)		
Implant	5 (1.7)		
Orthodontic	1 (0.3)		

^1^n (%)

Most incidents occurred after the administration of local anesthesia (*N* = 226, 75.3%); most occurred before the procedure (*N* = 118, 39.3%). Majority incidents occurred with a student operator, the highest with the undergraduate students (*N* = 184, 69.3%). Most incidents occurred in the clinical area (*N* = 275, 91.7%). The characteristics of the emergency incidents are shown in Table 1.

The symptoms experienced by the patients during the incidents were analyzed; for simplicity, patients with more than one symptom were narrowed down to the most relevant one. The most common symptom was lightheadedness (*N* = 155, 52%), followed by loss of consciousness (*N* = 88, 29%), and dyspnea (*N* = 31, 10%). Supplementary Table 1 reveals the frequencies of all symptoms.

The most common medical emergency diagnosis was syncope (*N* = 187 62.3%); eleven of these cases were transferred to the ER for either prolonged syncope or deterioration of vital signs. The second most common event was hypoglycemia (*N* = 36, 12%), three of which required further care in the ER. There were eight cases of panic attacks, one of which was transferred to the ER. The cases that improved in the dental clinic and did not require transfer to the ER for further intervention were postural hypotension (*N* = 7, 2.3%) and Epinephrine reaction (*N* = 10, 3.3%).

Table 2 shows a comparison of the diagnoses in patients with past medical history and those without any past medical history. The diagnoses for the entire cohort and for the pediatric patients (< 14 years) are shown in Table 3.

**Table 2:** The frequency of medical emergencies compared according to medical history

Diagnosis	No Medical History	Positive Medical History	Total = 300
Syncope	143	44	187
Hypoglycemia	23	13	36
Hyperventilation	10	4	14
Asthma	3	9	12
Epinephrine reaction	8	2	10
Panic Attack/Hysteria	4	4	8
Postural Hypotension	7	0	7
Seizure/Fit	5	1	6
Chest pain	0	6	6
Hypertensive Crisis	2	3	5
Hyperglycemia	0	3	3
Sodium Hypochlorite accident	2	0	2
Suspected Cardiovascular Accident	0	1	1
Allergic Reaction	1	0	1
Partial airway obstruction	1	0	1
Dehydration	1	0	1

^1^n (%)

**Table 3:** The diagnosis and disposition of the medical emergency events in the entire cohort (adults and pediatric patients)

***Entire Cohort (N = 300)***		
**Diagnosis**	**N** = **300**^**1**^	**Transfer to ER**
Syncope	187 (62.3)	11 (5.9%)
Hypoglycemia	36 (12)	3 (8.3%)
Hyperventilation	14 (4.6)	2 (15.4%)
Acute Asthma attack	12 (4)	4 (33.3%)
Epinephrine reaction	10 (3.3)	0
Panic attack/Hysteria	8 (2.7)	1 (12.5%)
Postural Hypotension	7 (2.3)	0
Seizure/Fit	6 (2)	2 (33.3%)
Chest pain	6 (2)	6 (100%)
Hypertensive crisis	5 (1.7)	3 (60%)
Hyperglycemia	3 (1)	2 (66.7%)
Sodium Hypochlorite accident	2 (0.7)	1 (50%)
Suspected Cardiovascular accident	1 (0.3)	1 (100%)
Allergic Reaction	1 (0.3)	1 (100%)
Partial airway obstruction	1 (0.3)	1 (100%)
Dehydration	1 (0.3)	1 (100%)
***Pediatric Patients < 17 (N = 12)***		
**Diagnosis**	**N** = **12**^**1**^	**Transfer to ER**
Syncope	4 (33.3)	0
Hypoglycemia	1 (8.3)	0
Hyperventilation	3 (25)	0
Acute Asthma attack	2 (16.6)	0
Epinephrine reaction	1 (8.3)	0
Seizure/Fit	1 (8.3)	0

^1^n (%)

Most patients recovered in the dental clinics (*N* = 261, 87%); those who recovered were mostly discharged without dental treatment after the incident (*N* = 182, 60.7%); nearly a quarter of them continued to receive dental treatment (*N* = 79, 26.3%). Whereas those who did not recover in the clinics (*N* = 39, 13%) were transferred to the ER, mostly via an ambulance (*N* = 27, 9%). Table 4 shows the post-incident clinical outcomes and patient disposition.

**Table 4:** Clinical outcome and disposition of the victims

Clinical Outcome	*N* = 300^1^
Recovery	261 (87)
No Improvement	39 (13)
Death	0
**Disposition**	
Sent Home	182 (60.7)
Continue Dental Treatment	79 (26.3)
Emergency Room	39 (13.0)
**Ambulance**	
Yes	27 (9.0)
No	273 (91.0)

^1^n (%)

**Fig. 1:**
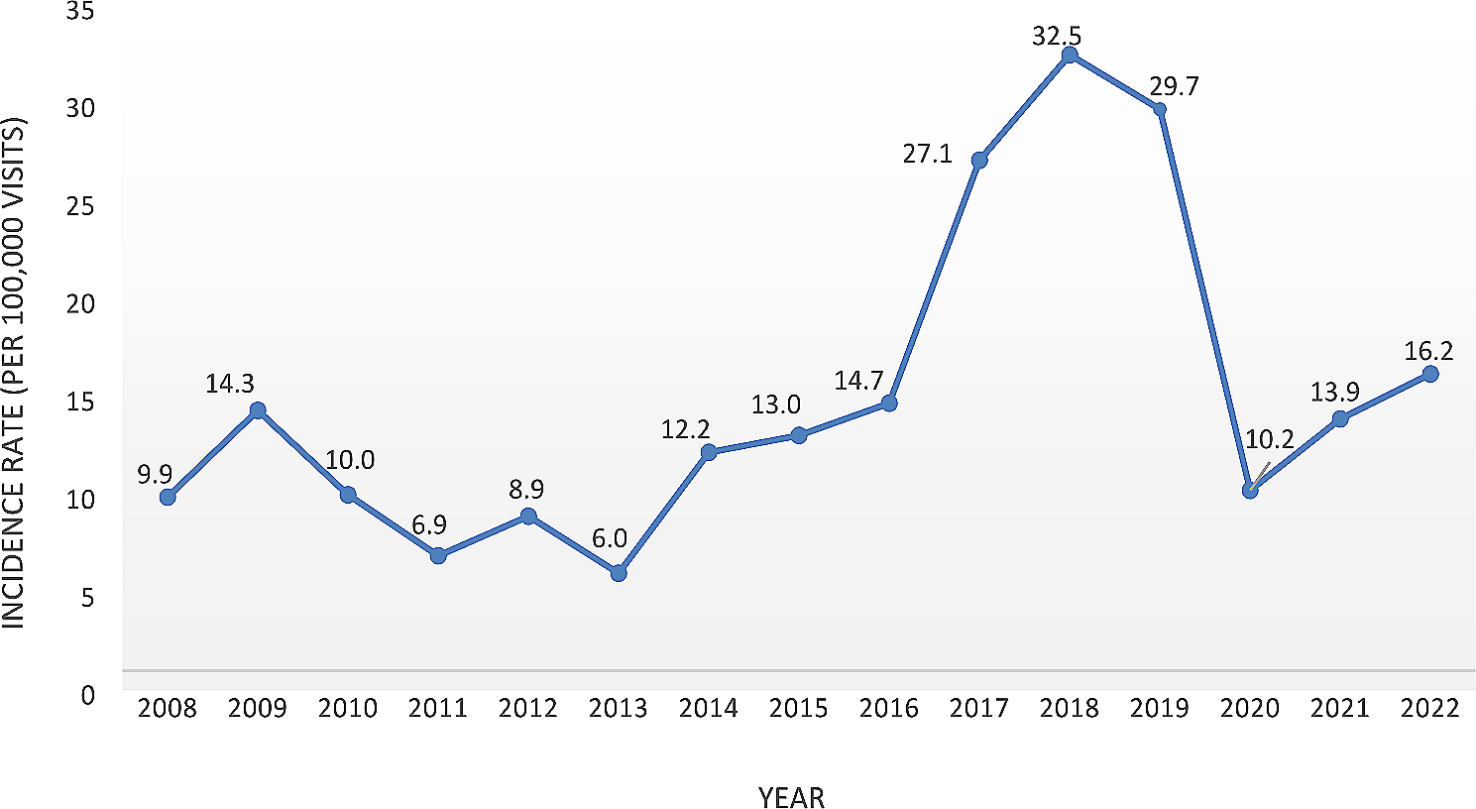
The incidence rate per year per 100,000 visits from 2008 to 2022

Overall, the incidence of emergency events in this study was 17.4 per 100,000. The annual incidence of events per 100,000 visits is visualized in Fig. 1. The incidence in the 14-year period ranged from 6 to 32.5 per 100,000 patients. Heightened incidence rates were observed during 2017–2019.

Supplementary Fig. 1 visualizes the number of cases by month; cases occurred most frequently in May and June. Numbers were overall lower in the Fall season.

During the study period, one of the serious and potentially life-threatening medical emergencies observed was a sodium hypochlorite accident, causing airway obstruction. The patient was a healthy 42-year-old woman who underwent root canal therapy on a mandibular anterior tooth. Following a one-hour irrigation with 3% sodium hypochlorite, the patient experienced extensive swelling in the lip and chin area, which rapidly spread to the submental and sublingual regions, resulting in upper airway obstruction [16]. Recognizing the severity and rapid progression of the case, prompt management was initiated while the patient was still in the dental clinic. She received 100% oxygen via facemask and 100 mg of intravenous hydrocortisone. Subsequently, she was immediately transferred to the emergency department, where she was intubated and then admitted to the intensive care unit for three days. After extubating, she was transferred to the ward for observation and eventually discharged in a stable and healthy condition. This case highlights the importance of following proper protocols, the availability of necessary medications, and effective communication with emergency medical services (EMS) for prompt transfer [16].

Another noteworthy case involved a 19-year-old woman who encountered two medical emergencies during a single dental visit. She presented to the dental clinic for the surgical removal of two third molars, displaying a high degree of anxiety. After providing verbal reassurance, local anesthesia was administered. However, the patient experienced pallor, complained of lightheadedness, and progressed into a vasovagal syncope episode. Immediately the patient was placed supine, and then airway, breathing, and circulation were assessed. Additionally, 100% oxygen was administered via a facemask. The patient regained consciousness and made a full recovery. While attempting to reassure the patient, the operator explained the situation, which caused her anxiety to resurface. This led to hyperventilation, manifested by an increased respiratory rate, and shortness of breath. Subsequently, she developed numbness and tingling in her hands. Appropriate measures were taken to calm the patient and guide her to breathe slowly into her cupped hands to regain control over her breathing rate and restore the balance between oxygen and carbon dioxide levels. The patient recovered well and was referred to a specialist to undergo the desired procedure under sedation or general anesthesia. This case emphasizes the importance of early recognition of anxiety in dental patients, discussing concerns and options before procedures, and formulating individual treatment plans to suit patient needs [17].

### Predictors of syncope

Owing to sample size limitations, not all characteristics could be incorporated into a single multivariable model. Therefore, all characteristics that had a *p*-value of < 0.2 were used in the multivariable model, which included procedure and ASA classification. The multivariate logistic regression model revealed that ASA classification 2 and 3 were associated with a 50% lesser likelihood of syncope compared to ASA classification 1 (OR = 0.54 [95% CI: 0.30–0.99], *p* = 0.049). Additionally, patients who did not undergo the procedure at the time of the incident were 4.22 times more likely to be associated with a syncope (OR = 4.22 [95% CI: 1.59–11.17], *p* = 0.004).

### Predictors of disposition

Undergoing a general dental procedure was associated with a 70% lesser likelihood of transfer to the ER compared to undergoing dental extraction and surgical procedures (OR = 0.32 [95% CI: 0.13–0.79], *p* = 0.01). An ASA classification of 2 or 3 was associated with a 3.56 times higher likelihood of transfer to the ER versus ASA classification 1 (OR = 3.56 [95% CI: 1.63–7.78], *p* = 0.001). Furthermore, a respiratory or other incident was 7.36 and 11.36 times more likely to be transferred to the ER, respectively (OR = 7.36 [95% CI: 2.09, 25.90], *p* < 0.001; and OR = 11.36 [95% CI: 4.41, 29.26], *p* = 0.002, respectively). The results from multivariable logistic regression models predicting syncope and disposition are presented in Table 5.

**Table 5:** Multivariable logistic regression models predicted syncope and disposition

Predictors of Syncope	OR (95%CI)	*p*-value
**Procedure**		
Extraction/Surgery	Reference	
General Dental procedures	0.61 (0.35, 1.06)	0.08
No Procedure	4.22 (1.59, 11.17)	**0.004**
**ASA Classification**		
1	Reference	
2, 3	0.54 (0.30, 0.99)	**0.049**
***Predictors of Disposition***	**OR (95%CI)**	***p-value***
**Procedure Group**		
Extraction/Surgery	Reference	
General Dental procedures	0.32 (0.13, 0.79)	**0.01**
No Procedure	2.64 (0.77, 9.07)	0.12
**ASA Classification**		
1	Reference	
2, 3	3.56 (1.63, 7.78)	**0.001**
**Diagnosis Group**		
Syncope	Reference	
Hypoglycemia	1.63 (0.40, 6.65)	0.49
Respiratory	7.36 (2.09, 25.90)	**0.002**
Other	11.36 (4.41, 29.26)	**< 0.001**

## Discussion

This was a 14-year retrospective study of 300 medical emergency incidents in an academic dental hospital in Saudi Arabia in a relatively younger and healthier cohort. The incidence of emergency events was 17.4 per 100,000 patients. Syncope was the commonest emergency; it was associated with ASA-1 and not undergoing a procedure. Overall, most incidents recovered, with only 13% requiring transfer to the ER. ASA classifications 2 and 3, dental extraction and surgical procedures, and a diagnosis of respiratory/other incidents predicted the transfer to the ER.

In this study, patients in the age group of 18–30 years had the highest frequency of medical emergencies (40%), which is younger than what Sooch et al. [12] reported (30–40 years). The mean age of this cohort was 34.2, whereas that reported by Anders et al. and D’Innocenzo et al. were much older (61 years and 49 years, respectively) [11, 14]. This difference could be accounted for by the younger Saudi population; 57.38% of the Saudi population are below 35 years in 2021 according to the General Authority for Statistics [18].

In this study, the majority (70%, *n* = 210) of patients were healthy, without comorbidities, unlike similar studies. Sorenson et al. reported that only 25% of the patients were healthy [10], while Obata et al. found comorbidities in 57% of the cases [4]. Moreover, patients with comorbidities are less likely to safely tolerate dental surgery-related stress [17].

The prevention of medical emergencies in the dental office warrants strict adherence to fundamental patient-safety principles: obtaining a detailed clinical history, pre-procedural examination of vital signs, including blood sugar, and patient selection based on clinical risk stratification by using an established scoring system, such as the ASA Patient Physical Status Classification [17]. Although the importance of proper medical history and vital signs in the prevention of medical emergencies is well established [17, 19], in our study, the highest number of medical emergency events occurred in younger patients without a medical history; the most frequent events in these 210 healthy patients were syncope (68%), followed by hypoglycemia (11%) and hyperventilation (5%). Moreover, patients who did not undergo a procedure were 4.22 times more likely to have a syncope, which emphasizes that some emergencies may be pre-procedural, particularly in anxious patients or in those with unstable medical conditions [7]. These results highlight the importance of stress reduction protocols that can assist in preventing medical emergencies in patients showing signs of fear and stress [17, 20]. In addition, having an adequate meal before appointments, establishing proper rapport with the patient to address concerns and alleviate anxiety, and reducing waiting and procedure times for high-risk patients is necessary for the prompt identification of emergencies [17]. Optimal premedication, procedural sedation, and post-operative pain control in accordance with the training and local policies can also help prevent medical emergencies in the dental office [17, 21].

At our institution, the patients are screened for medical problems, a thorough history and review of systems is undertaken, and appropriate referrals are conducted. The students are always supervised regarding the suitability of the case and any modifications in the dental treatment of the ASA-2 patients. The ASA-3 patients are treated by specialists/faculty for urgent dental procedures. One-third of our patients with medical emergencies were ASA-2 and − 3. The medical history was a predictor for the type of emergency; patients with a positive medical history (ASA 2 and 3 were less likely to report a syncope compared to ASA 1).

Retrospective practitioner surveys [3, 22, 23], and clinical records-based studies [4, 5] indicate that some medical emergencies are more common than others in a dental office: vasovagal syncope and hypoglycemia consistently figured among the top causes of medical emergency events in the majority of these studies; allergies, postural hypotension, dehydration, hyperventilation, and foreign body ingestion were also commonly reported in these studies, albeit less commonly than syncope and hypoglycemia. However, a study from the United States with a relatively older patient population reported hypertensive crisis as the most frequent emergency [13]. Thus, medical emergencies arising in the dental office may depend on the population characteristics.

In this study, the most common type of emergency was syncope, and it was the most common emergency in healthy ASA 1 patients. This is generally consistent with the literature. Obata et al. reported more than 60% of the incidents were vasovagal syncope [4]. In this study, due to the fine line between the diagnoses of syncope and presyncope, they were placed in one category because the diagnosis was established and characterized by a Nurse, EMT, and Dentist on-call, all of whom are not internists. Vasovagal syncope is the most common cause of loss of consciousness in the dental clinic secondary to anxiety [24], but in our cohort, 11 out of 187 cases of syncope were transferred to the ER due to prolonged syncope or instability in vital signs.

Hypoglycemia was the second most common emergency. Three of the 36 cases of hypoglycemia suffered from loss of consciousness and required further management in the ER. Diabetes mellitus (DM) is highly prevalent in Saudi Arabia. According to the International Diabetes Federation’s Atlas in 2021, 15.4% of adult Saudis aged 20–79 years have DM, the highest prevalence of DM among adults aged 20–79 years [25]. In a study by Al-Sebaei et al. [26], random blood sugar testing in dental outpatients revealed that 30.8% of high-risk patients had a blood glucose level above 200 mg/dL, i.e., they were potentially diabetic, undiagnosed, and unaware. Therefore, establishing a blood glucose level by a finger stick is incorporated in our school’s emergency protocol immediately after checking the airway, breathing, circulation, and vital signs; a glucometer is readily available in all emergency carts; students, faculty, and auxiliary staff are trained to use it. D50, D10, and glucagon are among the basic medications in our emergency carts.

Moreover, dentists have a crucial role in the prevention of diabetic emergencies in the dental clinic by measuring pre-procedure random blood glucose levels, which is particularly relevant in diabetic and high-risk patients undergoing dental treatment. In addition, dentists also play an important role in the referral of patients with abnormal blood glucose levels to internists [26].

There were eight cases of panic attack or hysteria; three of which were transferred to the ER. Only two patients had a history of psychiatric illness. Panic attacks in the dental setting have been reported by Sorenson [10], and although uncommon, they can still occur; this emphasizes the importance of patient selection, especially in the dental school setting, while considering modification of treatment to sedation techniques.

The majority of the study incidents occurred after the administration of local anesthesia, with 10 of these related to epinephrine reaction. An Epinephrine reaction is an adverse drug reaction that can occur with the intravascular injection of dental local anesthesia containing epinephrine. While it is generally not life-threatening, individuals with underlying cardiac conditions may experience a more severe reaction [27]. Dentists play a crucial role in promptly recognizing and managing patients who develop this condition. The symptoms result from the direct impact of epinephrine on the bloodstream, leading to a rapid heart rate and a sudden increase in blood pressure, with the primary symptoms being restlessness, headache, and shortness of breath. While the condition usually resolves on its own, dentists need to monitor the patient closely, and undertake appropriate measures to stabilize their condition. These cases are self-limiting [27], but the dentist needs to reassure the patient. The operator must monitor the patient closely and distinguish it from hyperventilation, panic attacks, or hypertensive crises. Consistent with the association of the majority of adverse effects with local anesthesia in the present study, a literature review revealed that dental local anesthesia can result in diverse adverse effects in 4.5–26.2% of cases undergoing dental procedures [28], many of which can present as medical emergencies, including allergic reactions, anaphylaxis, seizures, syncope, hypertensive crisis, tachycardia, and palpitations.

In the 14 years, 39 (13%) cases were transferred to the ER, 9% of them by ambulance, which is low compared to Anders et al. [11] (45%). It can be generally recommended that up to 90% of all emergencies arising in a dental office could be managed within the dental office; up to one-third could require an emergency consultation and day-care support; and about 10% could need hospitalization [4]. Timely contact with the ambulance services remains an integral part of the emergency preparedness plan of a dental office [29]. However, ambulance response times may not meet the internationally acceptable target of eight minutes in many countries [30]. For instance, in Saudi Arabia, the average ambulance response times were much higher in both urban and rural areas, which was 10.2 min even for Riyadh [31]. Therefore, although contacting an ambulance at the earliest is recommended, the possibility of delayed arrival of an ambulance must always be considered. Further, this reinforces the essential role of the dental team in providing the immediate first response, which can be life-saving, especially in the case of cardiac arrest when early CPR and even a 1-minute delay in CPR makes a big difference to the mortality [32]. However, our institution is connected to the main hospital, with access to an ambulance and EMT team that can be readily contacted and respond immediately.

Yet, mock drills to practice medical emergencies should be practiced periodically in dental facilities to ensure EMS teams have a timely response time and there are no obstacles in the way of the EMS. A fast response time amounts to faster medical attention and better prognosis.

In this study, all diagnoses had at least one case transferred to the ER except Epinephrine reaction and postural hypotension. Medical emergencies have the potential of getting worse and becoming serious and requiring transfer to an ER, contributing factors of which may be the patients’ underlying medical condition, the comfort level of the dentists and ER covering team, or the availability of emergencies equipment and drugs. This was confirmed in our study as the medical history (ASA 2 & 3) and the type of medical emergency were predictors for being transferred to the ER. The type of procedure performed also predicted the disposition of the patient. Patients undergoing a general dental procedure are less likely to be transferred to the ER compared to those undergoing a surgical procedure. Studies from Japan and Australia [4, 5] indicate that between 26.9% and 45.2% of all emergencies could be associated with dental extractions.

The incidence of medical emergencies in the dental office is not well-defined. In a 8-year retrospective study in a UDH in Japan [4], the incidence of medical emergencies was 0.0037%. In a 6-year retrospective study from an Australian academic dental institution [5], the incidence of medical emergencies was 0.037%. Our 14-year results are within the reported range at 0.017% (17.4 per 100,000).

The incidence of medical emergencies in this study fell in 2020 due to the Covid-19 pandemic. Remarkably, the cases increased in March, since the students must submit their final cases for final case discussion and grading in May and June before the final exams. Interestingly, medical emergencies declined in the first few months of the academic year, which corresponded to the beginning of patient treatment, such as screening and formulating treatment plans.

A medical emergency department was established in 2015, along with a taskforce, protocols, mock drills, and a full training module for students and faculty on an annual basis. A school-wide campaign was launched multiple times throughout 2016 to check, reinforce, and troubleshoot the system.

At the UDH, biannual mock drills are conducted with the primary goals of ensuring preparedness, practicing medical emergency protocols, assessing response times, testing communication systems, and evaluating the effectiveness of the medical emergency system. The mock drill scenario is planned and prepared by a dedicated mock drill committee. They develop a script that includes the patient symptoms and vital signs. The timing and details of the mock drill are not disclosed to the students, faculty, and supporting personnel involved. An actor is recruited, and the scenario is rehearsed to ensure a realistic simulation. After preparing the necessary equipment, the ideal location and time for the mock drill are selected within the UDH. During the execution of the mock drill, a member of the mock drill committee observes and documents response times, adherence to protocols, effective communication, and records any deficiencies identified within system. Following the drill, the nature of the exercise is disclosed, and a debriefing session is conducted with the participants. Observations and feedback are reviewed and discussed to identify areas for improvement, which is followed by a detailed report, focusing on response time, correct patient management, equipment availability or failure, and any identified training needs. Based on the report, medical emergency protocols are updated, training requirement are reassessed, and areas of deficiency are addressed with the UDH administration. These processes ensure that the facility, students, and personnel remain well-prepared and equipped to handle medical emergencies in the dental setting both effectively and efficiently.

There were zero deaths in our retrospective analysis. Although mortality statistics from dental emergencies are limited, the risk of death from a medical emergency arising in a dental office appears to be low (below 1%). In a 2020 study from China [33], out of 2,923 events, there were 6 (0.2%) deaths. In a 1999 British study [7], out of 2,287 emergency events, 20 deaths were recorded, again falling below 1%. Though the risk of death from medical emergencies in dental offices seems to be low, such unfortunate outcome can have major implications for dental professionals, including adverse media reporting, official enquiries, and/or litigation [34]. The British study [7] attributed all 20 deaths to cardiovascular events, such as cardiac arrest, myocardial infarction, and stroke (14, 4, and 2 deaths, respectively). A Japanese study also found that the causes of death for such deaths were mostly cardiovascular events [35]. However, an analysis of online reports [36] suggests that the cause of death could also be related to anesthesia, medication, or sedation, especially in younger patients.

### Limitations

The study uses recorded data and is retrospective. This needs to be considered when interpreting the results. However, we could not find any prospective research on this topic in the literature. Future research on this topic needs to consider and utilize the prospective study design. Moreover, this was a single-center study, like most of the previous studies on this topic; future studies need to involve multiple centers in their protocols. A systematic review of existing studies is also warranted to gain meaningful insights. Nevertheless, this single-center retrospective study was able to reveal the incidence and characteristics of medical emergencies in the dental clinics of the Middle East for the first time.

Even though the reports in the present study were written by certified registered nurses who had them reviewed and co-signed by doctors, there may still be some errors or discrepancies. This also holds true for potentially inaccurately documented confounding factors like past medical history. Furthermore, although certain associations between variables were identified in this work, causation between variables could not be demonstrated. Dental professionals and researchers need to recognize the importance of proper documentation and reporting of medical emergencies to facilitate the analysis and identification of associations between variables.

## Conclusion

In summary, we report an incidence of 0.017% (17.4 per 100,000) over a 14-year period. Although this incidence is low, dental practitioners should remain aware of the contributing factors, such as past medical history and anxiety. However, medical emergencies can occur in healthy individuals as well. Thus, preparation of the dental office, training of the personnel, and proper recording of the events are essential components of a well-established medical emergency protocol in dental institutions. The results of the present study can help inform and improve emergency response plans of dental offices, especially for those located in Saudi Arabia and Middle Eastern countries.

### Electronic supplementary material

Below is the link to the electronic supplementary material.

**Supplementary Figure 1**: The number of medical emergency events by month from 2008 to 2022 (N=300).
**Supplementary Table 1**: The frequencies of each symptom

## Data Availability

The entire deidentified dataset, data dictionary, and analytic code for this investigation are available upon request, from the date of article publication by contacting Maisa Al-Sebaei.

## Abbreviations

ACLS: Advanced cardiac life support
ASA: American Society of Anesthesiologists
BLS: Basic life support
CI: Confidence interval
CPR: Cardio-pulmonary resuscitation
DM: Diabetes mellitus
EMS: Emergency medical services
EMT: Emergency medical technician
ER: Emergency room
MERT: Medical emergency response team
OR: Odds ratio
RN: Registered nurse
SCFHS: Saudi commission for health specialties
SD: Standard deviation
UDH: University dental hospital
