# Supplementary material for: Frequency and features of medical emergencies at a teaching dental hospital in Saudi Arabia: a 14-year retrospective observational study
Source: BMC Emerg Med. 2024 Mar 13;24:41. doi: 10.1186/s12873-024-00957-4 (PMC10935771; doi:10.1186/s12873-024-00957-4)
Supplement: Supplementary file 1 — Supplementary Figure 1: The number of medical emergency events by month from 2008 to 2022 (N=300). Supplementary Table 1: The frequencies of each symptom [file 12873_2024_957_MOESM1_ESM.docx]

**Supplementary Figure 1**

**Supplementary Table 1:** The frequencies of each symptom

| ***Symptom*** | ***N = 300****^1^* |
| --- | --- |
| Lightheadedness | 155 (52%) |
| Loss of consciousness | 88 (29%) |
| Dyspnea | 31 (10%) |
| Shivering | 20 (6.7%) |
| Headache | 13 (4.3%) |
| Chest Pain | 8 (2.7%) |
| Numbness | 7 (2.3%) |
| Anxiety | 6 (2.0%) |
| Heart Palpitation | 5 (1.7%) |
| Cold Extremities | 5 (1.7%) |
| Seizure | 5 (1.7%) |
| Sweating | 4 (1.3%) |
| Nausea | 4 (1.3%) |
| Restlessness | 3 (1.0%) |
| Facial Swelling | 3 (1.0%) |
| Blurred Vision | 2 (0.7%) |
| Vomiting | 2 (0.7%) |
| Wheezing | 2 (0.7%) |
| Slurred Speech | 1 (0.3%) |
| Weakness | 1 (0.3%) |
| Fever | 1 (0.3%) |
| Muscle Spasm | 1 (0.3%) |
| ^1^n (%) |  |
